# Low-calorie sweeteners in the human diet: scientific evidence, recommendations, challenges and future needs. A symposium report from the FENS 2019 conference

**DOI:** 10.1017/jns.2020.59

**Published:** 2021-01-25

**Authors:** Alison M. Gallagher, Margaret Ashwell, Jason C. G. Halford, Charlotte A. Hardman, Niamh G. Maloney, Anne Raben

**Affiliations:** 1 Nutrition Innovation Centre for Food and Health (NICHE), Biomedical Sciences Research Institute, Ulster University, Coleraine BT52 1SA, Northern Ireland, UK; 2 Ashwell Associates, Ashwell, Hertfordshire, UK; 3 School of Psychology, University of Leeds, Leeds, UK; 4 Department of Psychology, University of Liverpool, Liverpool, UK; 5 Department of Nutrition, Exercise and Sports, Faculty of Science, University of Copenhagen, Rolighedsvej 30, Frederiksberg DK-1958, Denmark

**Keywords:** Low-energy sweeteners, Intense sweeteners, Non-nutritive sweeteners, Weight management, Glycaemic control, Nutrition policy

## Abstract

Overconsumption of free sugars, particularly from sugar-sweetened beverages (SSB), has potential negative health impacts. Implementation of a range of public health strategies is needed to reduce intakes of free sugars, including reducing portion sizes, promoting healthier dietary choices and reformulating foods and beverages. Although low-calorie sweeteners (LCS) are a useful tool for reducing energy intake and control glucose response when consuming sweet foods and drinks, several opinions persist about the adverse health effects of LCS, many of which are based on poor, little or no scientific evidence. This symposium report summarises key messages of the presentations and related discussions delivered at a scientific symposium at the 13th European Nutrition Conference (FENS 2019). These presentations considered the scientific evidence and current recommendations about the use and potential benefits of LCS for human health, with a particular focus on current evidence in relation to body weight and glycaemic control. Many of the studies to date on LCS have focused on low-calorie sweetened beverages (LCSB); however, the psychological and behavioural factors influencing consumer beliefs and consumption of LCSB need to be further explored. Current recommendations for LCS use are described, including the conclusions from a recent expert consensus report identifying the challenges that remain with LCS research. Finally, existing knowledge gaps and future actions are described, as well as two large ongoing research projects: SWITCH and SWEET.

## Graphical abstract

**Figure:**
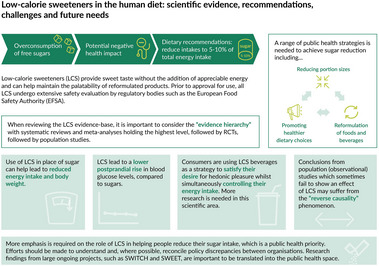

## Introduction

Free sugars, which encompass all monosaccharides and disaccharides added to foods by the manufacturer, cook or consumer, and sugars that are naturally present in honey, syrups, fruit juices and fruit juice concentrates^(1,2)^ have recently drawn particular attention in relation to public health. The potential negative impact of overconsumption of free sugars, particularly from sugar-sweetened beverages (SSB), in relation to weight gain, increased risk of type II diabetes mellitus and tooth decay is well recognised and has informed dietary recommendations to reduce population intakes of free sugars to 10 or sometimes 5 % of the total energy intake^(1,2)^. However, given the current high intakes of free sugars, achieving such reductions is challenging and will require implementation of a range of public health strategies, including reducing portion sizes, promoting healthier dietary choices and reformulating foods and beverages^(3)^.

Non-nutritive sweeteners provide a desired sweet taste without the addition of appreciable energy and can help maintain the palatability of reformulated products. They can be broadly categorised as bulk sweeteners or intense sweeteners. This symposium report will focus on intense sweeteners, commonly referred to as low-calorie sweeteners (LCS), and it will consider the scientific evidence for the use and potential benefits of LCS.

### Safety evaluation

**Fig. 1.**
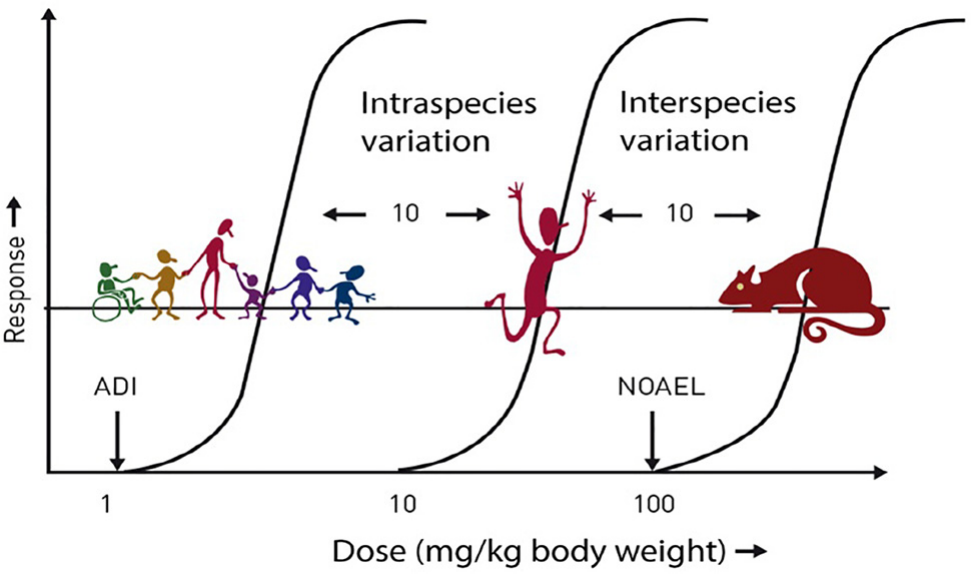
Safety factors applied to establish the ADI. ADI, acceptable daily intake; NOAEL, no observed adverse effect level. ADI is typically set at 1/100th of the NOAEL (allowing for 10-fold reduction for intraspecies variation and 10-fold reduction for interspecies variation). Source: Logue et al.^(4)^

Prior to approval for use, all LCS undergo extensive safety evaluation; the responsibility for these evaluations lies with regulatory bodies such as the European Food Safety Authority (EFSA), the FAO/WHO Joint Expert Committee on Food Additives (JEFCA) and the US Food and Drug Administration (FDA). These evaluations usually result in the establishment of the Acceptable Daily Intake (ADI) for each LCS. The ADI is typically calculated following the application of large safety factors (often a factor of 100 times lower than the ‘no observed adverse effect level’ (NOAEL) to give a large margin of safety for even the most susceptible and sensitive individuals in the population, including children and pregnant women (Fig. 1). The ADI is often misinterpreted; it does not represent a threshold between safe and unsafe, but it refers to a lifetime exposure situation, not a single occasion, and, therefore, infrequent consumption of levels higher than the ADI is not a health concern^(5)^.

At present within the EU, a total of eleven LCS are approved for use (Table 1) in accordance with the EU Regulation 1333/2008^(6)^ on food additives; the use of LCS in most of the cases is authorised in beverage or food categories with a reduction of at least 30 % of beverage/food product energy or with no added sugars. Replacing free sugars in SSBs with LCS is relatively straightforward and as such offers the potential for ‘sugar-swaps’ in SSB consumers. However, under EU Regulation 1333/2008, the permitted use of LCS depends on the food category/categories into which the product falls, and, currently, LCS cannot be incorporated into most of the fine baked products (e.g. biscuits or cakes), thus potentially limiting the opportunities for food reformulation^(7)^. Furthermore, whilst LCS may be a useful tool for reducing energy intake and control glucose response when consuming sweet foods and drinks, several theories persist about the adverse health effects of LCS acutely or in the long term, many of which are based on poor, little or no scientific evidence^(3)^.

**Table 1.** LCS approved for use in Europe

LCS	E-number	Year of first EU safety evaluation and approval	Sweetness^a^	ADI (mg/kg/BW)
Saccharin and its salts	E954	1977	300–500	5
Aspartame	E951	1984	180–200	40
Acesulfame-K (Ace-K)	E950	1984	200	9
Cyclamates	E952	1984	30	7
Thaumatin	E957	1984	2000–3000	ADI not specified
Neohesperidine dihydroalcone	E959	1988	1900	5
Aspartame–acesulfame salt	E962	2000	350	As for aspartame and Ace-K
Sucralose	E955	2000	600	15
Neotame	E961	2007	7000–13 000	2
Steviol glycosides	E960	2010	300	4
Advantame	E969	2013	37 000	5

a Sweetness relative to sucrose; LCS, low-calorie sweeteners; ADI, Acceptable Daily Intake; BW, body weight.

Source: Adapted from Logue *et al.*^(4)^

### Recommendations from health-related organisations

In 2011, the EFSA^(8)^ concluded that there is sufficient scientific information to support the claims that intense sweeteners, as all sugar replacers, lead to a lower postprandial rise in blood sugar levels, if consumed in place of sugars. Public Health England^(9)^ recommends ‘sugar-swaps’, replacing sugary soft drinks for diet, sugar-free or no added sugar varieties to reduce free sugar intakes. This position has also been endorsed by those at the British Dietetic Association,^(10)^ who highlight that swapping SSB for low-calorie sweetened beverages (LCSB) is likely to be beneficial for most individuals from a weight management, dental and diabetes perspective; however, they highlight that healthier drink options/alternatives should be actively encouraged (e.g. milk-based drinks) as these provide additional nutritional benefits that LCSB do not. Given that weight management is key to managing (and preventing) type II diabetes, the BDA also advocates the use of LCS in adults and children, where this is in place of free sugars, noting that in such cases LCS may be a useful means of reducing energy intake and can help maintain a healthy body weight^(10)^. In 2018, Diabetes UK launched a Position Statement^(11)^, which concluded that low or non-caloric sweeteners are shown to be safe and that they can be used as part of a strategy for adults and children in the management of weight and diabetes.

More recently, the American Diabetes Association (ADA) also issued a new Consensus Report in 2019^(12)^. Some of the conclusions were that replacing added sugars with sugar substitutes (LCS) could decrease the daily intake of carbohydrates and calories. These dietary changes could beneficially affect glycaemic, body weight and cardiometabolic control. The ADA also stated that using sugar substitutes does not make an unhealthy choice healthy; rather, it makes such a choice less unhealthy^(12)^. Finally, if sugar substitutes are used to replace caloric sweeteners, without caloric compensation, they may be useful in reducing caloric and carbohydrate intake, although further research is needed to confirm these concepts^(12)^.

The American Academy of Pediatrics has recently released a policy statement^(13)^ on the use of LCS in children, noting in their key findings and recommendations that when substituted for sugar-sweetened foods or beverages, LCS can reduce weight gain or promote weight loss (albeit to a small extent) in children (and adults) but recognising that data are limited. They found no absolute contraindications to LCS use in children and, for some affected by certain conditions (e.g. obesity, type I and type II diabetes), there may be a benefit for the use of LCS if substituted for nutritive sweeteners (namely free sugars)^(13)^. The only exception is the use of aspartame and neotame in children with phenylketonuria (PKU), since both these LCS contain phenylalanine which cannot be metabolised by those with PKU^(13)^.

## Outline of this symposium report

This symposium report summarises key messages from presentations and related discussions delivered at a scientific symposium at the 13th European Nutrition Conference (Federation of Nutritional Societies (FENS 2019), which considered the scientific evidence and recommendations for the use and potential benefits of LCS for human health, with a particular focus on current evidence in relation to body weight and glycaemic control. To date, many of the studies on LCS have focused on LCSB; however, the psychological and behavioural factors influencing consumer beliefs and consumption of LCSB need to be further explored. Current recommendations for LCS use are described, including the conclusions from a recent expert consensus report highlighting the challenges that remain with LCS research. Finally, existing knowledge gaps and future actions, as well as two large ongoing research projects: SWITCH and SWEET, are described.

## LCS as a means for weight and glycemic control: outcomes of systematic reviews and meta-analyses

**Professor Anne Raben (University of Copenhagen**) started by discussing general considerations when reviewing the LCS evidence base.

When considering reviews or original studies in health science, it is important to remember the ‘evidence hierarchy’ with systematic reviews and meta-analyses holding the highest level, followed by randomised controlled trials (RCT) and then by population studies. This should always be taken into account when studying and interpreting results from different studies. Thus, conclusions from population studies may suffer from the ‘reverse causality’ phenomenon whereby, for example, individuals with overweight/obesity may choose to consume LCS foods and drinks to reduce their risk of weight gain and not *vice versa*^(14)^. Furthermore, data on glycaemic outcomes may be confounded by changes in body weight^(15)^. Additionally, assessments of LCS intakes often consider only certain sources of LCS (e.g. LCSB) and/or LCS as a homogenous group despite differing biological fates^(5)^. This has the potential of not adequately capturing intakes of individual LCS or allowing for a reliable estimation of overall LCS intakes^(3)^.

The design of RCTs should also be carefully considered. In this aspect, especially the fixed-calorie vs the *ad libitum* study design is crucial. For example, if using a fixed-calorie design, it is not possible to show how a certain dietary component, in this case LCS, may influence appetite, food intake or body weight in the long term, since appetite regulation has been taken out of the equation. In contrast, an *ad libitum* study design will be able to demonstrate whether a certain LCS increases, decreases or has no effect on appetite, food intake, glycaemic control or body weight compared with a control situation (e.g. sucrose or water). In this situation, a study participant will be able to eat until satiation is reached and is not obliged to eat a specific, predefined amount of dietary energy.

It is also important to recognise that LCS have different chemical structures and, therefore, different metabolisms in the human body^(5)^. Due to these differences, LCS have very different digestion and uptake patterns in the gut, and their metabolic effects in the human body are, therefore, also likely to be very different^(16)^. Furthermore, it should be remembered that LCS have a very high sweetness intensity and are, therefore, usually consumed in extremely small amounts compared with nutritive sweeteners such as sucrose. Thus, any physiological effect is likely to be minimal at most.

### LCS as a means for body weight control: the evidence

Professor Raben then focused on LCS and weight control. There have been several studies on the effectiveness of the sweeteners, mainly aspartame, on weight control, and the very early studies were summarised by de la Hunty *et al*.^(17)^.

A study by Raben's group using a 10-week *ad libitum* design demonstrated that body weight and fat mass decreased significantly in adults with overweight after the intake of foods and drinks containing LCS compared with similar products containing added sugar (sucrose), resulting in an average difference in body weight of 2⋅6 kg^(18)^. Ten years later, a 1⋅5-year study in children (aged 4–11 years) also showed a very clear picture^(19)^. In the present study, participants received 250 ml/d of an LCSB or a similar SSB while at school and the LCSBs reduced body weight gain and fat accumulation compared with the sugar-containing beverages. In both these studies, the volunteers were blinded to the intervention arms.

Comprehensive reviews and meta-analyses were published a few years later by Miller and Perez^(20)^ and by Rogers *et al*.^(21)^. The review by Rogers *et al*.^(21)^ was the first to consider both acute and longer-term animal, human, cross-over, RCT and cohort studies on LCS, appetite, energy intake and body weight regulation. A meta-analysis of short-term RCTs (129 comparisons) showed a significantly reduced *ad libitum* consumption (94 kcal) after intake of LCS *v*. sugar-sweetened foods or beverages, with no difference when compared with water. A meta-analysis of intervention RCTs ranging from 1 to 40 months showed that LCS *v.* sugar led to a reduction in body weight of 1⋅35 kg (nine comparisons), and a similar relative reduction in body weight *v*. water (three comparisons). The systematic review from Azad *et al*.^(22)^ was neutral for RCTs showing no benefit nor weight gain in the LCS groups compared with the controls. Toews *et al*.^(23)^ concluded from their systematic review, which included thirty-five observational studies, that ‘In adults, evidence of very low and low certainty from a limited number of small studies indicated a small beneficial effect of NSSs [i.e. LCS] on body mass index’ and ‘For all other outcomes, no differences were detected between the use and non-use of NSSs, or between different doses of NSSs.’

In some reviews, the different LCS have been considered separately, which is very relevant, given that LCS have different metabolic fates. Recent reviews on aspartame alone or steviol glycosides alone showed no significant differences in body weight between the LCS and a control or sucrose^(24,25)^. Such analyses are, however, hampered by the limited number of studies, and the majority of RCTs have used a combination of sweeteners. More recently, a 12-week RCT included four different LCSBs (aspartame, saccharin, sucralose and rebaudioside A) and sucrose in a parallel-arm design including 154 participants^(26)^. The beverages contained 400–560 kcal/d (sucrose treatments) or <5 kcal/d (LCS treatments). The results showed that sucrose and saccharin led to significantly increased body weight (by 1⋅85 and 1⋅18 kg, respectively) when compared with aspartame, rebaudioside A and sucralose. The change in body weight observed was directionally negative and significantly lower with sucralose when compared with the three other LCS (weight difference ≥ 1⋅37 kg). Energy intake also decreased with sucralose, supporting the observed weight loss in this group^(26)^.

Water is generally believed to be the preferred choice over LCS beverages. To clarify this, the role of LCS in comparison with water was investigated in a 1-year RCT with 303 people with overweight and obesity. All participants took part in a 12-week behavioural weight loss programme and were then assigned to either 710 ml of water or LCSBs per day for 1 year. Convincingly, the study showed that participants drinking LCSBs maintained more than twice the weight loss (6⋅2 kg) compared with participants drinking water (2⋅6 kg), i.e. an improvement of weight control with LCS compared with water^(27)^.

To summarise, the balance of evidence indicates that the use of LCS in place of sugar (sucrose), in children and adults, can lead to reduced energy intake and body weight. The current evidence, although still limited, points to no difference or similar effects when LCS are compared with water.

### LCS as a means of glycaemic control: the evidence

Professor Raben then summarised the studies on LCS and glycemic control. In a 10-week *ad libitum* study, postprandial glucose (PPG) and insulin concentrations after 10 weeks were significantly lower after LCS vs sucrose^(28)^. This was also the case after adjusting for differences in body weight changes and fasting values at week 10. After further adjusting for differences in energy and sucrose intake, postprandial insulin (PPI) was still significantly lower on the LCS diet. The possible effect of LCS on gastrointestinal hormones (e.g. GIP and GLP-1) was also investigated. The results showed that postprandial GIP and GLP-1 concentrations at 10 weeks were significantly lower after LCS compared with sucrose, even after adjusting for differences in body weight changes, fasting GIP and GLP-1 values, energy and sucrose intake^(28)^.

An RCT investigating the effect of 0, 350 or 1050 mg aspartame/d in a beverage for 12 weeks reported no differences in glucose, insulin, GLP-1 or GIP during an oral glucose tolerance test (OGTT) in healthy, lean adults^(29)^. A similar result for glucose and insulin was seen during an OGTT after a 6-month intake of either 1 Diet Coke, Regular Coke, water or milk^(30)^. In a systematic review and meta-analyses, LCS were also not found to elevate blood glucose level – rather a gradual decline in glucose was seen after LCS consumption^(31)^. It was also seen that the glycaemic impact of LCS did not differ by the type of LCS (aspartame, saccharin, steviosides and sucralose), but to some extent by age, body weight and diabetes state. These latter factors are, therefore, important to consider when comparing results from different studies on LCS.

Two other systematic reviews on LCS and glycaemia were published in the past 5 years^(32,33)^. Based on fourteen observational studies, LCS seemed to be related to the development of metabolic diseases, but adiposity was often an important confounder. In twenty-eight clinical trials, contradictory results were seen and, furthermore, studies were not comparable^(32)^. Another analysis of forty-one studies showed that some LCS triggered physiological responses, although this was inconsistent. Without co-ingestion of carbohydrates, LCS acted similarly to water, and with co-ingestion of carbohydrates, LCS reduced plasma glucose compared with sucrose^(33)^. Very recently, a review and meta-analyses on steviol glycosides reported no effect of LCS on glucose^(25)^.

Professor Raben took the opportunity to present some preliminary results from a systematic review and meta-analysis of human intervention studies. It examined the acute effect of LCS intake on PPG and PPI responses and found that the ingestion of LCS has no acute effects on the mean change in postprandial glycemic or insulinemic responses compared with a control intervention^(34)^.

Professor Raben concluded her presentation by looking to the future and describing the Horizon-2020 project ‘SWEET’ (www.sweetproject.eu, 2018–2023, Grant Agreement No. 774293), which aims to dig further into the potential risks and benefits of sweeteners and sweetness enhancers (S&SEs). The focus is on health, obesity, safety and sustainability in a multidisciplinary approach. A core part of SWEET is a 2-year RCT across Europe, which will investigate the effect of the prolonged use of S&SEs in a whole healthy diet approach (foods and drinks) on diet compliance, weight control and obesity-related risk factors (e.g. glycaemia and lipidemia) and safety (e.g. gut microbiota and allergenicity) in both adults and children.

More specifically, SWEET consists of a consortium of twenty-nine pan-European research, consumer and industry partners. In different work packages, diverse S&SE containing products will be developed and new databases generated (health, technological and sweetness). The potential toxicity and the regulatory frameworks affecting S&SE use will be assessed. In short to medium-term studies, the impact of specific S&SEs alone or in combination pertaining to gut hormone release, microbiota, central nervous system response, eating behaviour, satiety, reward, cravings and food choice in differing populations (gender, BMI and weight status) will be investigated. Besides the large-scale 2-year RCT, epidemiological evidence is being re-examined using multiple data sets with up to 170,000 individuals across different European populations. A comprehensive analysis of dietary composition and urinary biomarkers will be done to validate self-reported S&SE intake. The environmental, social and economic sustainability of increasing the production of S&SEs through life cycle analysis will also be investigated in the 5-year project.

## Psychological and behavioural factors influencing consumer beliefs and consumption of LCS beverages

The second speaker was **Professor Jason Halford (University of Leeds)**, who looked at consumer attitudes to LCS beverages and summarised studies looking at psychological and behavioural factors influencing their consumption.

Consumption of LCS beverages (LCSB) is often higher among adults who are overweight or have obesity, compared with adults of a healthy weight^(35)^. For example, studies from the United States have shown that females who had obesity were more likely to consume LCSBs compared with individuals who were of a healthy weight and males^(36,37)^. As noted previously, evidence from systematic reviews and meta-analyses suggests that any association with obesity is more likely due to reverse causality,^(20,21)^ and a possible explanation for this association is that individuals with overweight and obesity may utilise LCSBs in response to their excess adiposity and/or weight gain, rather than vice versa^(38)^. Indeed, recent studies suggest that LCSB consumption is tied to consumer efforts to decrease their energy intake and, in particular, the intake of nutritive sweeteners^(39,40)^. Drewnowski and Rehm^(41)^ reported an association between the intention to lose weight and LCSB use and found that previous weight fluctuations were a predictor of LCSB consumption. Frequent use of LCSBs is also associated with dietary restraint and weight concerns compared with non-habitual use^(42)^. Taken together, these findings are consistent with the notion that individuals with a high BMI often use LCSBs as a strategy to restrict energy intake in order to control their body weight^(43,44)^. A more detailed understanding is needed of consumers’ attitudes and beliefs towards LCSBs. The psychological mechanisms underpinning the observed effect of LCSB on energy intake also need to be further elucidated.

### Attitudes and beliefs about LCSBs

To address current gaps in knowledge, a questionnaire on the *Attitudes and Beliefs towards LCS Beverages* has recently been developed and this quantifies key factors associated with LCSBs by measuring attitudes and beliefs associated with their consumption^(45)^. In this research, frequent consumers were defined as individuals who consumed over 825 ml of LCSBs per day, as determined using a self-reported online Food Frequency Questionnaire (see Appleton and Conner^(42)^). Results indicated that frequent and non-consumers of LCSBs had polarised attitudes and beliefs towards LCSBs^(45)^. Specifically, frequent consumers had more positive beliefs that LCSBs were palatable and effective in controlling their appetite and body weight in comparison with non-consumers. As such, beliefs about hedonic enjoyment and health appear to influence consumer decisions about consumption or avoidance of LCSBs. These contrasting beliefs, and the more negative views among non-consumers, are not surprising, given that several studies have raised public awareness of potential adverse health effects of LCSBs,^(36,46–48)^ which likely discourages their consumption among some consumers. The importance of overcoming such misinformation and scepticism about LCS will be discussed in further detail later in the paper.

In line with these findings, Catenacci *et al*.^(49)^ examined the motivations behind the consumption of LCSBs in individuals who had successfully maintained weight loss. They found that 78 % of consumers believed that LCSBs helped them control or reduce their total calorie consumption whilst also avoiding weight gain. In addition, palatability was another important factor driving the consumption of LCSBs in this sample. This is further evidence that LCSBs may help people to control their appetite and satisfy their food cravings when dealing with the continuous challenge of maintaining weight loss over time. Taken together, goals concerning body image and weight, coupled with positive hedonic reward and palatability beliefs, appear to be significant factors in motivating the consumption of LCSBs.

### Psychological mechanisms underpinning the effect of LCSB on energy intake

As noted previously, in a systematic review and meta-analysis of short-term experimental studies, energy intake was significantly reduced when foods or beverages containing LCS were consumed relative to their sugar-containing counterparts^(21)^. However, the psychological mechanisms that underpin the effect of LCSB on energy intake are unclear.

One possibility is that consumers are using these beverages as a strategy to satisfy their desire for hedonic pleasure whilst simultaneously controlling their energy intake. As established earlier, frequent consumers of LCSB typically have higher levels of restrained eating, and as such, their eating behaviour is likely to be characterised by cycles of food restriction and disinhibited eating, ultimately making them more susceptible to weight gain^(50,51)^. According to the goal conflict model of eating behaviour, individuals with high dietary restraint find it difficult to regulate their food intake because they are juggling two conflicting goals: the hedonic goal of eating enjoyment whilst also satisfying the longer-term goal of weight maintenance^(52)^. These goals frequently conflict with each other, because low-energy ‘diet’ foods are often less hedonically pleasing than foods of higher energy content^(53)^. Consumption of LCSBs may play an important role in this context. Sweet-tasting foods and beverages are hedonically pleasing to many people and LCSBs may, therefore, satisfy food cravings and hedonic eating goals. In addition, due to their very low energy content, LCSBs may simultaneously preserve weight control goals, thereby realigning previously conflicting goals in the goal conflict model. In this respect, LCSBs may offer great potential as a means of managing hedonic food motivations and cravings in individuals who struggle with their weight.

To explore this idea, a recent study used a ‘chocolate craving’ manipulation to examine the effect of priming hedonic eating goals on *ad libitum* energy intake in frequent consumers and non-consumers of LCSBs^(54)^. It was hypothesised that energy intake would be greater after the hedonic eating prime relative to a control prime in non-consumers, but that frequent LCSB consumers would be protected from this effect (due to their consumption of LCSBs satisfying their hedonic eating motivations). Findings from two experiments did not consistently support this hypothesis. However, in the second experiment, the frequent consumers ingested fewer calories (less energy intake) overall when LCSBs were available relative to a condition when they were unavailable. Furthermore, when LCSBs were unavailable, frequent consumers reported lower perceived behavioural control (i.e. lower self-efficacy), lower meal enjoyment and higher eating-related guilt relative to the condition when LCSBs were available. These findings suggest that LCSBs assist frequent consumers in exercising self-control over food choices and weight control. This is important, given that previous research has found that emotions, such as eating-related guilt, can lead to negative outcomes including selection of indulgent foods, increased food consumption and long-term weight gain^(55,56)^.

Interestingly, Maloney *et al*.^(54)^ also found that frequent LCSB consumers had a visual attentional bias towards images of LCSBs, relative to water and SSB. This finding suggests that frequent consumers view LCSBs as hedonically desirable. This attentional bias was not evident for non-consumers, which is consistent with research showing that individuals selectively attend to personally relevant environmental stimuli^(57,58)^. Importantly, frequent consumers showed a visual preference for LCSBs relative to sugar-containing beverages, which indicates a specific bias towards LCSBs rather than a general bias towards sweet-tasting products. This finding is contrary to the hypothesis that LCSBs encourage a generalised preference for sweet-tasting foods^(59–61)^. Indeed, evidence to date suggests that exposure to sweet taste does not promote a subsequent preference for sweet products but, in fact, leads to a *reduced* preference for sweetness in the short term^(62)^. However, the authors of this systematic review highlighted that the existing evidence base is weak and that there is a need for longer-term, adequately powered studies.

Collectively, findings to date indicate that LCSBs appear to be fulfilling a psychological role for consumers by satisfying their hedonic food motivations without violating dieting goals. In doing so, LCSBs could play a meaningful role in reducing energy intake by facilitating self-regulation in the face of high-calorie food temptation, without the accompanying caloric intake and guilt. Nevertheless, further research is still needed to understand how these beverages affect cognitions and subsequent appetitive behaviours.

Professor Halford ended his presentation by emphasising that, whilst the beneficial effects of LCS beverages in weight management have been reported^(27,63)^, the effects of LCS over longer time periods need to be further elucidated. The SWITCH trial (Trial registration: Clinical Trials: NCT02591134; registered: 23 October 2015) is addressing these research gaps by exploring the longer-term effects of LCSBs in weight management and underpinning physiological and psychological mechanisms^(64)^. Specifically, this ongoing trial is assessing the effect of LCSBs, relative to water, on both short and long-term weight management, as well as examining several candidate behavioural and biological mechanisms (e.g. changes in glycaemic control, fasting lipid profiles, appetite, energy intake, food choice, mood and attitudes) through which these effects may arise relative to water. This research will provide a new and detailed understanding of the role that LCSBs play in weight loss and maintenance, particularly their psychological impact, and of the behavioural mechanisms that mediate these effects.

## Identified research gaps on LCS and suggested future actions

The final speaker was **Dr Margaret Ashwell (Ashwell Associates)**, who summarised the outcomes from an Expert Consensus Workshop on LCS which was held in November 2018^(65)^. The aims of this workshop were to identify the reliable facts on LCS, suggest research gaps and propose future actions. During the workshop, seventeen experts (the panel) discussed three themes identified as key to the science and policy of LCS: (1) weight management and glucose control; (2) consumption, safety and perception; (3) nutrition policy. In brief, the panel agreed that the safety of LCS is demonstrated by a substantial body of evidence reviewed by regulatory experts. Current levels of consumption, even for high users, are within agreed safety margins. However, the panel identified that better risk communication is needed^(65)^.

The panel identified research gaps for each of the three Themes (summarised in Table 2)^(65)^. In summary, the panel's conclusions were that the substantial body of evidence concerning LCS safety should be communicated in a consistent manner. More emphasis is required on the role of LCS in helping people reduce their sugar and energy intake, which is a public health priority. The panel also felt that efforts should be made to understand and, where possible, reconcile policy discrepancies between organisations and reduce regulatory hurdles that impede product development and reformulation designed to reduce free sugars and/or energy intake. For example, the requirement in the EU that the use of LCS in foods/beverages in most cases should reduce the energy content of the given food/beverage by 30 % limits the options available for a more modest reformulation or stepwise reduction in free sugar content of food/beverages.

**Table 2.** Consensus statements on research gaps identified by the expert consensus panel

Theme 1 – Role of LCS in weight management and glucose control
1. What are the long-term effects of LCS on glucose tolerance, gut function, cardiometabolic effects, gut microbiota and weight management?
2. How are these effects altered according to personal factors, such as age, sex, ethnicity, socio-economic status, health status, diet and lifestyle?
3. How do these effects differ according to the dietary context (*ad lib v*. weight-control diet) and form of LCS (in liquids or solids), and the type or blend of LCS?
4. Does reducing exposure to sweetness have consequences for food choice and intake in the medium-to-long term?
5. Can LCS help improve long-term type II diabetes management when they are a part of standard dietary and lifestyle approaches?
Theme 2 – Consumption and safety of LCS and consumer perception
1. Which factors (including knowledge, attitudes and behaviours) influence consumer perception of the risks and benefits of LCS consumption? Are these the same for health professionals?
2. There is a need for in-depth data relating to the current patterns of LCS consumption at multiple levels, and across countries and regions, to strengthen the evidence base.
3. There is a need for more reliable measures of LCS exposure, such as biomarkers. Further development of these and better linkage of food composition and dietary databases are needed to help monitor the changing use and consumption of LCS.
Theme 3 – Role of LCS in relation to nutrition policy
1. Can LCS help individuals meet the population level dietary recommendations for reduction of sugar intake (e.g. to 5 % (average) or 10 % (for individuals))? If so, how can this be achieved?
2. How does a dietary approach that includes LCS-sweetened foods and drinks affect dietary quality compared with low-sugar diets?
3. What are the best strategies to communicate LCS safety and efficacy to interested parties such as health professionals and the general public?

Source: Adapted from Ashwell *et al.*^(65)^

The consensus statements and recommendations arising from the Expert Consensus Workshop^(65)^ should serve to assist policymakers and other stakeholders including non-governmental organisations (NGOs), health professionals, research funding bodies and the food and beverage industry.

## Symposium discussion

Professor Halford was asked about the different results obtained when solid foods *v.* liquids were sweetened with LCS. Do they have the same effects on appetite, intake behaviour, etc.? He replied that this is not yet known and drew attention to the fact that this was identified as one of the research gaps (Table 2) in the consensus report^(65)^.

The panel was asked about the safety of LCS for consumption by pregnant women and children. The panel referred to the decisions of the regulatory authorities and noted previously that all currently available LCS have been extensively evaluated for their safety and for each LCS the ADI level is a conservative estimate acceptable dietary intake over an entire lifetime and is inclusive of all age groups and sensitive sub-populations, including children and pregnant women^(5)^.

One questioner referred to the recently published EPIC cohort study^(66)^ where a greater consumption of total, sugar-sweetened and artificially sweetened soft drinks was associated with a higher risk of all-cause mortality. The panel, as well as other experts in the audience, pointed out the flaws of observational studies and limitations of their designs, including the potential biases in self-reported intake assessment methods, residual confounding and reverse causation.

Another question related to the fact that LCS are a chemically diverse group of compounds. Do they have the same effects on outcomes such as appetite, food intake, the gut microbiome, weight control, etc.? Should they be examined individually or as a group? The panel commented that often, when the outcome under consideration is on the effects of reducing free sugars, they are treated as a single group (without consideration of their heterogeneous nature). However, the recent research on the gut microbiome emphasised that, especially in this respect, individual differences could be very important although, at typical levels of consumption, no adverse effect on human health via gut microbiome had been established for any LCS^(67,68)^. Furthermore, it is worth noting that whilst a few studies have shown changes in the gut microbiome, they have been mainly in rodent studies (e.g. Suez *et al*.^(69)^) and have considered supraphysiological doses with no relevance to realistic human intakes. Also, the recent study on body weight from the group of Richard Mattes emphasises that different LCS can have different effects on body weight^(26)^.

The final question was about recommendations to healthcare professionals about the use of LCS by children. The panel referred the questioner to the recent policy statement by the American Academy of Pediatrics with a set of recommendations and guidance for paediatricians^(13)^.

## Conclusions and future research needs

Given the current public health interest on the impact of free sugars on human health and the potential contributions that LCS could play in achieving current recommendations for intakes of free sugars, it is important that the research findings from projects such as SWITCH and SWEET are fully realised and translated into the public health space. However, it is also important to recognise that even with a well-developed evidence base, translation of any benefit of LCS into wider public health benefits will ultimately be hampered if scepticism surrounding LCS use persists within the general public, health professionals and other key stakeholders. As highlighted by Ashwell *et al*.^(65)^, a better understanding of the differing views on the risks and benefits of LCS among experts, policymakers and the general public should be prioritised. Countering misinformation, where appropriate, is needed to ensure a balanced reporting of the public health relevance of the totality of the research evidence base^(70)^. This should be done together with public health messages which boost public understanding of LCS, focusing on their safety and the appropriate use of LCS within the context of a healthy diet. As a next step, the identified research gaps (as outlined earlier) and suggested future actions represent a blueprint for the way forward^(65)^. A focus on better risk communication and on the potential benefits of LCS will assist policymakers and other stakeholders including NGOs, health professionals, research funding bodies and the food and beverage industry. This will support wider public health strategies aimed at reducing excessive intakes of free sugars and thereby result in positive impacts on human health.
